# The risk factors of future exacerbations and treatment responses among different inhalation therapies of patients with preserved ratio impaired spirometry

**DOI:** 10.7189/jogh.16.04070

**Published:** 2026-02-20

**Authors:** Jun Cao, Tian Sun, Huan Yang, Lijie Zhou, Qin Shen, Ling Lin, Tao Li, Ping Zhang, Yuqin Zeng, Ping Chen, Qing Song, Si Lei, Jianmin Li

**Affiliations:** 1 Department of Pulmonary and Critical Care Medicine, Hunan Provincial People's Hospital and the first-affiliated hospital of Hunan normal university, Changsha, Hunan, China; 2 Clinical Medicine Research Center For Respiratory Rehabilitation, Changsha, Hunan, China; 3 Department of Pulmonary and Critical Care Medicine, the Second Xiangya Hospital of Central South University, Changsha, Hunan, China; 4 Research Unit of Respiratory Disease, Central South University, Changsha, Hunan, China; 5 Clinical Medical Research Center for Pulmonary and Critical Care Medicine in Hunan Province, China; 6 Diagnosis and Treatment Center of Respiratory Disease, Central South University, Changsha, Hunan, China; 7 Department of General Medicine, The Second Xiangya Hospital of Central South University, Changsha, Hunan, China

## Abstract

**Background:**

Preserved ratio impaired spirometry (PRISm) is closely related to chronic obstructive pulmonary disease (COPD). However, there is a lack of relevant research on the treatment of patients with PRISm. Therefore, this study aimed to investigate the risk factors of future exacerbations and treatment responses among different inhalation therapies of patients with PRISm.

**Methods:**

This is a retrospective cohort study. Patients with PRISm were registered in the real-world study on the status of diagnosis and treatment of COPD (RealDTC) study between January 2017 and August 2024. Data on demographics, pulmonary function, symptom scores, number of exacerbations and hospitalisations in the past year, inhalation therapy regimens including long-acting muscarinic antagonist (LAMA), long-acting β2-agonist (LABA) + inhaled corticosteroid (ICS), LABA + LAMA, and LABA + LAMA + ICS, and comorbidities were collected. The number of exacerbations, frequent exacerbations, hospitalisations, and all-cause of mortality were collected during one year of follow-up.

**Results:**

A total of 575 patients were included for the final analysis. During one year of follow-up, 144 (25.0%) patients experienced exacerbations. The patients experienced exacerbations had higher age, symptom score, number of exacerbations and hospitalisations in the past year, as well as higher proportion of biofuel exposure and without inhalation therapy. Logistic regression analysis showed that age, number of hospitalisations in the past year, and without inhalation therapy were the independent risk factors for patients experienced exacerbations. Furthermore, after propensity score matching, the patients without inhalation therapy had higher number of exacerbations, frequent exacerbations, and hospitalisations during one year of follow-up. However, there were no significant differences in future exacerbations, frequent exacerbations, hospitalisations, and all-cause of mortality among LAMA, LABA + LAMA, LABA + ICS, and LABA + LAMA + ICS.

**Conclusions:**

Patients with PRISm had high risk of future exacerbations. Inhalation therapy could reduce the risk of future exacerbations and clinicians should recommend mono-LAMA to patients with this condition.

Preserved ratio impaired spirometry (PRISm) is a common pulmonary dysfunction that is closely related to chronic obstructive pulmonary disease (COPD), multiple comorbidities and other risk factors. The PRISm is not always a stable status and may develop into COPD over time [1,2]. Therefore, early diagnosis and treatment of patients with PRISm are crucial for preventing and treating COPD.

The Global Initiative for Chronic Obstructive Lung Disease (GOLD) 2024 report defined PRISm in detail and proposed that PRISm patients are at risk of developing airflow obstruction [3]. Consequently, this comorbidity subgroup has garnered increasing clinical focus. Currently, researches on patients with PRISm have predominantly centred on their prevalence, risk factors, and clinical outcomes [4–6]. Nevertheless, PRISm is strongly associated with increased respiratory symptoms [7]. Cough, sputum production, and dyspnea are prevalent respiratory manifestations in patients with PRISm. Those presenting with respiratory symptoms further demonstrate significantly elevated risks of gastrointestinal symptoms and cardiovascular complications [8,9]. In addition, exacerbations are important deterioration events for patients with chronic respiratory diseases [10]. Previous studies have shown that patients with PRISm have a risk of future exacerbations [11]. Consequently, early intervention in this population is clinically significant for reducing symptoms and the risk of exacerbations. Inhalation therapy – including long-acting β_2_-agonists (LABA), long-acting muscarinic antagonists (LAMA), and inhaled corticosteroids (ICS) – serve as a first-line therapy for COPD patients to alleviate symptoms and reduce the risk of exacerbations [3]. However, their therapeutic efficacy in patients with PRISm remains incompletely elucidated.

Therefore, this study aimed to investigate the risk factors of future exacerbations and treatment responses among different inhalation therapies of patients with PRISm.

## METHODS

### Study participants

This was a retrospective cohort study. The patients were registered in the real-world study on the status of diagnosis and treatment of COPD (RealDTC) study between January 2017 and August 2024, as described previously [12]. They had been diagnosed with PRISm according to the GOLD 2024 report [3]: the ratio of forced expiratory volume in one second to forced vital capacity (FEV1/FVC) was ≥ 0.7, and the forced expiratory volume in one second percentage predicted (FEV1%pred) was < 0.8 after inhaling a bronchodilator. Patients with active tuberculosis, asthma, and severe heart, liver or kidney disease were excluded from this study.

This study was approved by the ethic review board of the Second Xiangya Hospital of Central South University and conducted in accordance with the Declaration of Helsinki (2016076). All patients provided written informed consent.

### Data collection

Data on age, sex, education level, body mass index (BMI), smoke history, smoking (packs/y), biofuel exposure, FEV1%pred, FEV1/FVC, COPD assessment test (CAT) scores, modiﬁed Medical Research Council (mMRC) scores, number of exacerbations and hospitalisations in the past year, comorbidities including chronic heart disease, hypertension, lung cancer, diabetes, and bronchiectasis, and inhalation therapy regimens including LAMA, LABA + LAMA, LABA + ICS, LABA + LAMA + ICS, short-acting β_2_-agonists (SABA), and short-acting muscarinic antagonists (SAMA) were recorded for patients first visited hospitals. The clinicians provided inhalation therapy regimens based on the patient’s condition.

All patients completed one year of follow-up to collect data on exacerbations, frequent exacerbations, hospitalisations, and all-cause of mortality. The patients lost to follow-up were excluded from this study.

### Sample size calculation

The sample size was calculated using PASS 15.0 in the part of confidence interval (CI) for one proportion. We used the pre-experiment exacerbation incidence rate (24.9%) as the assumed sample proportion, a confidence level of 0.95, and a two-sided CI. Accounting for a 20% dropout rate, the minimum required sample size was calculated to be 556.

### Variable definition

Exacerbation was defined as a condition requiring antibiotics, oral corticosteroids, or hospitalisation [13]. Frequent exacerbation was defined as two or more exacerbations per year [14]. Biofuel exposure was defined as the use of biomass fuels for cooking or heating for at least two hours per day for at least one year [15]. A former smoker has had ≥ 10 packs/y but has not smoked for more than six months, while a current smoker has had smoking exposure of ≥ 10 packs/y [16].

Prescription outcomes, including adjusting treatment, were defined as changing the inhalation therapy drugs or stopping them for more than three months during the one year of follow-up [17].

### Statistical analysis

The Student’s *t*-test was used to analyse continuous variables with normal distributions and homogeneity of variance, and the results are expressed as mean ± standard deviation. Otherwise, the variables are expressed as median and interquartile range (IQR) and non-parametric tests was used to analyse data. The χ^2^ test or Fisher exact test was used to analyse categorical variables. Multivariate logistic regression was used to assess the relative factors for patients with PRISm who experienced exacerbations during follow-up; the variables were listed in Table 1 (*P* < 0.05); the odds ratio (OR) and 95% CI were calculated. Propensity score matching (PSM) between those who used inhalation therapy and those who without inhalation therapy was conducted using *R* package version 2.15.3 (R Foundation for Statistical Computing, Vienna, Austria), with a ratio 4:1. A value of *P* < 0.05 was considered statistically significant. For statistical analysis we used the SPSS 26.0 (IBM, Armonk, USA) and Free Statistics software version 1.7.1 (Beijing Fengrui Technology Co., Ltd, Beijing, China).

**Table 1:** The clinical characteristics of the patients suffering from exacerbation during follow-up

Variables	Non-exacerbation, (n = 431)	Exacerbation, (n = 144)	*P*-values
**Age in years, x̄ ± SD**	63.3 ± 10.3	65.3 ± 8.4	0.032*
**Sex, n (%)**			0.902
Male	312 (72.4)	105 (72.9)	
Female	119 (27.6)	39 (27.1)	
**Education level, n (%)**			0.304
Under junior high school	339 (78.7)	119 (82.6)	
Over high school	92 (21.3)	25 (17.4)	
**BMI (kg/m^2^), x̄ ± SD**	24.1 ± 4.1	23.6 ± 4.4	0.280
**Smoke history, n (%)**			0.666
Never smoker	175 (40.6)	61 (42.4)	
Former smoker	80 (18.6)	30 (20.8)	
Current smoker	176 (40.8)	53 (36.8)	
**Smoking, packs/y (MD, IQR)**	24.0 (0.0, 40.5)	20.0 (0.0, 40.0)	0.439
**Biofuel exposure, n (%)**			<0.001*
Yes	147 (34.1)	75 (52.1)	
No	284 (66.6)	69 (51.8)	
**Pulmonary function (x̄ ± SD)**			
FEV1%pred	69.7 ± 11.8	67.5 ± 11.3	0.050
FEV1/FVC	75.0 ± 5.3	75.0 ± 4.4	0.951
**CAT scores (x̄ ± SD)**	13.0 ± 6.6	13.7 ± 5.8	0.254
**CAT scores, n (%)**			0.020*
<10	140 (32.5)	32 (22.2)	
≥20	291 (67.5)	112 (77.8)	
**mMRC scores (MD, IQR)**	1 (1, 2)	2 (1, 2)	0.028*
**mMRC scores, n (%)**			0.170
0–1	229 (53.1)	67 (46.5)	
≥2	202 (46.9)	77 (53.5)	
**Therapy, n (%)**			<0.001*
LAMA	95 (22.0)	13 (9.0)	
LABA+LAMA	63 (14.6)	14 (9.7)	
LABA+ICS	118 (27.4)	28 (19.4)	
LABA+LAMA+ICS	95 (22.0)	29 (20.1)	
SABA or SAMA	2 (0.5)	1 (0.7)	
No inhalation	58 (13.5)	59 (41.1)	
**Comorbidities, n (%)**			
Chronic heart disease	20 (4.6)	7 (4.9)	0.914
Hypertension	26 (6.0)	8 (5.6)	0.834
Lung cancer	7 (1.6)	1 (0.7)	0.686
Diabetes	11 (2.6)	1 (0.7)	0.311
Bronchiectasis	34 (7.9)	9 (6.2)	0.517
**Exacerbations in the past year (MD, IQR)**	0 (0, 1)	1 (0, 2)	0.020*
**Exacerbations in the past years, n (%)**			0.018*
0	236 (54.8)	60 (41.7)	
1	93 (21.6)	44 (30.6)	
≥20	102 (23.7)	40 (27.7)	
**Hospitalisations in the past year (MD, IQR)**	0 (0, 1)	0 (0, 1)	<0.001*
**Hospitalisations in the past year, n (%)**			<0.001*
0	311 (72.2)	76 (52.8)	
≥1	120 (27.8)	68 (47.2)	

BMI – body mass index, COPD – chronic obstructive pulmonary disease, CAT – COPD assessment test, FEV1% pred – forced expiratory volume in one second percentage predicted, FEV1 – forced expiratory volume in one second, FVC – forced vital capacity, ICS – inhaled corticosteroid, IQR – interquartile range, LAMA – long-acting muscarinic antagonist, LABA – long-acting β2-agonist, MD – median, mMRC – modiﬁed medical research council, SABA – short-acting β2-agonist, SAMA – short-acting muscarinic antagonist, x̄ – mean

*Statistically significant results.

## RESULTS

### The clinical characteristics of the patients

**Figure 1:**
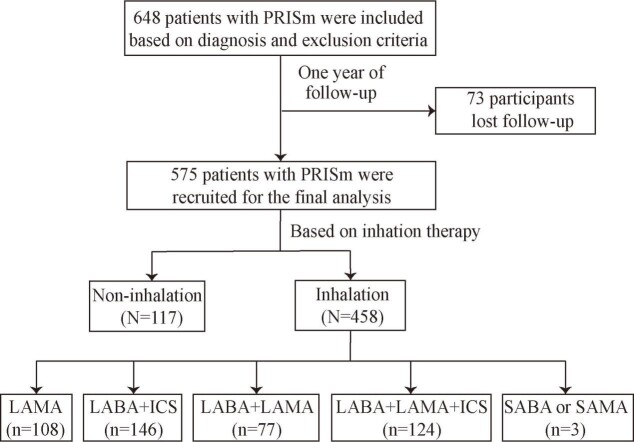
Flowchart. ICS – inhaled corticosteroid, LAMA – long-acting muscarinic antagonist, LABA – long-acting β2-agonist, PRISm – preserved ratio impaired spirometry, SAMA – short-acting muscarinic antagonist, SABA – short-acting β2-agonist.

A total of 648 patients were included based on diagnosis and exclusion criteria. During one year of follow-up, 73 patients lost to follow-up. A total of 575 patients were recruited for the final analysis (**Figure 1**).

The mean age was 63.8 ± 9.9 years, with males accounting for 72.5%. A total of three (0.5%) patients received SABA or SAMA, 108 (18.8%) received LAMA, 77 (13.4%) received LABA + LAMA, 146 (25.4%) received LABA + ICS, and 124 (21.6%) received LABA + LAMA + ICS, while 117 (20.3%) patients without inhalation therapy (Table S1 in the **Online Supplementary Document**). During one year of follow-up, there were 144 (25.0%) patients experienced exacerbations, 65 (11.3%) experienced frequent exacerbation, and 77 (13.4%) experienced hospitalisations. There were 101 (22.1%) patients adjusted inhalation therapy during one year of follow-up (Table S2 in the **Online Supplementary Document**). However, for the patients without inhalation therapy at baselines did not add inhalation therapy during follow-up.

### The clinical characteristics of the patients experienced exacerbation during follow-up

Patients experienced exacerbation had higher age, mMRC scores, number of exacerbations and hospitalisations in the past year. In addition, these patients had a higher proportion of CAT scores ≥20, biofuel exposure, and without inhalation therapy (*P* < 0.05) (**Table 1**).

Multivariate logistic regression analysis showed that age (OR = 1.026; 95% CI = 1.004–1.049), number of hospitalisations in the past year ≥1 (OR = 2.186; 95% CI = 1.427–3.350), and without inhalation therapy (OR = 8.382; 95% CI = 4.117–17.066) were the independent risk factors for patients experienced exacerbations (*P* < 0.05) (**Table 2**).

**Table 2:** Multivariate analysis of relative factors for exacerbation in patients*

Variables	OR	95% CI	*P*-values
Age	1.026	1.004–1.049	0.020†
Biofuel exposure			
*No*	Reference		
*Yes*	1.903	1.246–2.906	0.003†
Hospitalisations in the past year			
*0*	Reference		
*≥1*	2.186	1.427–3.350	<0.001†
Therapy			
*LAMA*	Reference		
*LABA + LAMA*	1.783	0.769–4.145	0.177
*LABA + ICS*	1.851	0.892–3.841	0.099
*LABA + LAMA + ICS*	1.781	0.854–3.714	0.124
*SABA or SAMA*	3.070	0.242–38.949	0.387
*No inhalation*	8.382	4.117–17.066	<0.001†

COPD – chronic obstructive pulmonary disease, CAT – COPD assessment test, OR – odds ratio, CI – confidence interval, ICS – inhaled corticosteroid, LAMA – long-acting muscarinic antagonist, LABA – long-acting β2-agonist, SAMA – short-acting muscarinic antagonist, SABA – short-acting β2-agonist.

*Adjusted for CAT scores and exacerbation in the past year.

†Statistically significant results.

### The future exacerbation between inhalation and non-inhalation during one year of follow-up after PSM

After PSM, 111 patients without inhalation therapy and 444 patients with inhalation therapy were analysed for future exacerbations. The baseline clinical characteristics showed no significant differences between the two groups (Table S3 in the **Online Supplementary Document**).

Furthermore, compared with patients who used inhalation therapy, the patients without inhalation therapy had a higher number of exacerbations, frequent exacerbations, and hospitalisations during one year of follow-up. However, there was no significant difference in all-cause of mortality between the two groups (**Table 3**).

**Table 3:** The future exacerbation between inhalation and non-inhalation during one year of follow-up after propensity score matching

Variables	PSM		*P*-values
	**Non-inhalation (n = 111)**	**Inhalation (n = 444)**	
Exacerbations during one year (MD, IQR)	0 (0, 2)	0 (0, 0)	<0.001*
Exacerbations, n (%)			<0.001*
*Yes*	55 (49.5)	85 (19.1)	
*No*	56 (50.5)	359 (80.9)	
Frequent exacerbations, n (%)			<0.001*
*Yes*	29 (26.1)	34 (7.7)	
*No*	82 (73.9)	410 (92.3)	
Hospitalisations during one year (MD, IQR)	0 (0, 1)	0 (0, 0)	<0.001*
Hospitalisations, n (%)			<0.001*
*Yes*	30 (27.0)	44 (9.9)	
*No*	81 (73.0)	400 (90.1)	
All-cause of mortality, n (%)			0.589
*Yes*	0 (0.0)	4 (0.9)	
*No*	111 (100.0)	440 (99.1)	

MD – median, IQR – interquartile range

*Statistically significant results.

### The treatment responses of different inhalation therapies in patients with PRISm

After adjusting for confounding factors including age, sex, BMI, smoke history, biofuel exposure, FEV1%pred, FEV1/FVC, CAT scores, mMRC scores, exacerbations in the past year, prescription outcomes, and comorbidities, there were no significant differences in future exacerbations, frequent exacerbations, hospitalisations, and all-cause of mortality among LAMA, LABA + LAMA, LABA + ICS, and LABA + LAMA + ICS (Table S4–6 in the **Online Supplementary Document**).

## DISCUSSION

COPD is a common, preventable and treatable disease; however, the widespread underdiagnosis and misdiagnosis lead to patients receiving improper or no treatment [3]. The GOLD report is the most widely accepted guideline among clinicians for the management and treatment of COPD. According to the GOLD report, PRISm patients will not remain in this state indefinitely. Evidence indicates that the majority of patients with PRISm patients transition to normal spirometry, whereas the remainder maintain PRISm status or progress to COPD [3]. Notably, PRISm constitutes an independent risk factor for the development of airflow limitation [18–20].

Exacerbations, as important deterioration events for patients, can accelerate pulmonary function decline and disease progression. In fact, Wallström et al. [21] found that, with follow-up extended to a median of 1.90 years for moderate exacerbations, the exacerbation rate for PRISm patients was 0.17 events per patient-year. When the follow-up was extended to a median of 1.96 years, the hospitalisation rate for PRISm patients was 0.22 events per patient-year. Yoon et al. [11] found that the annual incidence of moderate-to-severe exacerbation was 0.56 in patients with PRISm. This was similar to our results. In our study, 25.0% of patients with PRISm experienced exacerbations during one year of follow-up. In addition, we identified several independent risk factors for patients with PRISm experienced exacerbations, including age and hospitalisations in the past year. This implies that for PRISm patients of advanced age and those with a history of hospitalisations, implementing targeted therapeutic strategies to mitigate the risk of future exacerbations is imperative.

In addition, we found that without inhalation therapy was an independent risk factor for patients with PRISm experienced exacerbations. Furthermore, we found that patients treated with inhalation therapy had a lower risk of future exacerbations, frequent exacerbations, and hospitalisations. This implies that it is necessary to provide inhalation therapy for patients with PRISm to decrease the risk of future exacerbations. However, there was no significant difference in all-cause of mortality between the two groups. This might be related to the short follow-up period and the relatively low mortality rate. In addition, the patients without inhalation therapy had a higher risk of future exacerbations which may reflect systematic differences rather than treatment effect.

Inhalation therapy, including LAMA, LABA + LAMA, LABA + ICS, and LABA + LAMA + ICS, is the most commonly used treatment for patients [22]. Therefore, we analysed treatment responses among different inhalation therapies for patients with PRISm and found no significant differences in future exacerbations, frequent exacerbations, hospitalisations, and all-cause of mortality among LAMA, LABA + LAMA, LABA + ICS, and LABA + LAMA + ICS. This implies that mono-LAMA may be sufficient to reduce the risk of exacerbations in patients with PRISm, avoiding excessive medical treatment in the real-world.

This study has limitations. First, although this is a multicentre study, the sample size is relatively small. Therefore, a larger sample size will be required in future research. Second, a relatively small number of patients with PRISm used mono-SABA or SAMA, and LABA. Therefore, we cannot determine whether mono-SABA, SAMA, or LABA had efficacy similar to mono-LAMA in reducing future exacerbations. Third, this study had a relatively short follow-up period. Long-term follow-up observation of changes in pulmonary function is also particularly important. Fourth, this is a real-world study, randomised controlled trials (RCT) need to be investigated in the future. Fifth, we explicitly state that confounding by indication persists despite PSM adjustment, and that our findings should be interpreted as associative rather than causal. We further suggest that future confirmatory studies should adopt RCT designs. In addition, the heterogeneity of PRISm may exert a critically influence on treatment response. Finally, we are not sure whether it is health care access, socioeconomic status, or prescribing patterns, pulmonary function trajectories over 12 months were correlated with future exacerbations.

## CONCLUSIONS

Patients with PRISm had high risk of future exacerbations. Inhalation therapy could reduce the risk of future exacerbations and clinicians should recommend mono-LAMA to patients with this condition.

## Additional material


Online Supplementary Document
