# Supplementary material for: The risk factors of future exacerbations and treatment responses among different inhalation therapies of patients with preserved ratio impaired spirometry
Source: J Glob Health. 2026 Feb 20;16:04070. doi: 10.7189/jogh.16.04070 (PMC12922469; doi:10.7189/jogh.16.04070)
Supplement: Online Supplementary Document [file jogh-16-04070-s001.pdf]

Supplement to: Cao J, Sun T, Yang H, Zhou L, Shen Q, Lin L, Li T, Zhang P, Zeng Y, Chen P, Song Q, Lei S, Li J. The risk factors of future exacerbations and treatment responses among different inhalation therapies of patients with preserved ratio impaired spirometry. J Glob Health. 2026;16:04070.

Supplement table 1. The clinical characteristics of the patients.

Variables	Total (n=575)
Age (years), (Mean ± SD)	63.8 ± 9.9
Sex, n (%)	
Male	417 (72.5)
Female	158 (27.5)
Education level, n (%)	
Under junior high school	458 (79.7)
Over high school	117 (20.3)
BMI (kg/m^2), (Mean ± SD)	23.9 ± 4.1
Smoke history, n (%)	
Never smoker	236 (41.0)
Former smoker	110 (19.1)
Current smoker	229 (39.9)
Smoking, (packs/year) (Median, IQR)	24.0 (0.0, 40.0)
Biofuel exposure, n (%)	
Yes	222 (38.6)
No	353 (61.4)
Pulmonary function, (Mean ± SD)	
FEV1 %pred	69.1 ± 11.7
FEV1/FVC	75.0 ± 5.1
CAT scores, (Mean ± SD)	13.2 ± 6.4
CAT scores, n (%)	
<10	172 (29.9)
≥20	403 (70.1)
mMRC scores, (Median, IQR)	1 (1, 2)
mMRC scores, n (%)	
0-1	296 (51.5)
≥2	279 (48.5)
Therapy, n (%)	
LAMA	108 (18.8)
LABA+LAMA	77 (13.4)
LABA+ICS	146 (25.4)
LABA+LAMA+ICS	124 (21.6)
SABA or SAMA	3 (0.5)
No inhalation	117 (20.3)
Comorbidities, n (%)	
Chronic heart disease	27 (4.7)

Hypertension	34 (5.9)
Lung cancer	8 (1.4)
Diabetes	12 (2.1)
Bronchiectasis	43 (7.5)
Exacerbations in the past year, (Median, IQR)	0 (0, 1)
Exacerbations in the past years, n (%)	
0	296 (51.5)
1	137 (23.8)
≥20	142 (24.7)
Hospitalizations in the past year, (Median, IQR)	0 (0, 1)
Hospitalizations in the past year, n (%)	
0	387 (67.3)
≥1	188 (32.7)

Abbreviations: BMI, Body Mass Index; COPD, Chronic Obstructive Pulmonary Disease; CAT, COPD Assessment Test; FEV1 %pred, Forced Expiratory Volume in one second percentage predicted; FEV1, Forced Expiratory Volume in one second; FVC, Forced Vital Capacity; ICS, Inhaled Corticosteroid; IQR, InterQuartile Range; LAMA, Long-Acting Muscarinic Antagonist; LABA, Long-Acting β2-Agonist; mMRC, modified Medical Research Council; SAMA, Short-Acting Muscarinic Antagonist; SABA, Short-Acting β2-Agonist.

Supplement table 2. The future exacerbation of the patients during one year of follow-up.

Variables	Total (n=575)
Exacerbations during one year, (Median, IQR)	0 (0, 1)
Exacerbations, n (%)	
Yes	144 (25.0)
No	431 (75.0)
Frequent exacerbations, n (%)	
Yes	65 (11.3)
No	510 (88.7)
Hospitalizations during one year, (Median, IQR)	0 (0, 0)
Hospitalization, n (%)	
Yes	77 (13.4)
No	498 (86.6)
All-cause of mortality, n (%)	
Yes	4 (0.7)
No	571 (99.3)
Prescription outcomes, n (%)	
Adjust treatment	101 (22.1)
Continuous using	357 (77.9)

Abbreviations: IQR, InterQuartile Range.

Supplement table 3. The clinical characteristics of the patients between inhalation and non-inhalation after propensity score matching.

Variables	PSM		P - values
	Non-inhalation (n=111)	Inhalation (n=444)	
Age (years), (Mean ± SD)	62.4 ± 9.3	64.2 ± 9.8	0.090
Sex, n (%)			0.059
Male	90 (81.1)	321 (72.3)	
Female	21 (18.9)	123 (27.7)	
Education level, n (%)			0.754
Under junior high school	89 (80.2)	350 (78.8)	
Over high school	22 (19.8)	94 (21.2)	
BMI (kg/m^2), (Mean ± SD)	23.5 ± 4.2	24.1 ± 4.1	0.176
Smoke history, n (%)			0.126
Never smoker	35 (31.5)	185 (41.7)	
Former smoker	23 (20.7)	87 (19.6)	
Current smoker	53 (47.8)	172 (38.7)	
Smoking, (packs/year) (Median, IQR)	30.0 (0.0, 43.8)	22.0 (0.0, 40.0)	0.162
Biofuel exposure, n (%)			0.599
Yes	44 (39.6)	164 (36.9)	
No	67 (60.4)	280 (63.1)	
Pulmonary function, (Mean ± SD)			
FEV1 %pred	70.9 ± 11.0	69.1 ± 11.2	0.135
FEV1/FVC	75.0 ± 4.7	75.0 ± 5.2	0.962
CAT scores, (Mean ± SD)	12.6 ± 6.7	13.3 ± 6.1	0.294
CAT scores, n (%)			0.353
<10	37 (33.3)	128 (28.8)	
≥20	74 (66.7)	316 (71.2)	
mMRC scores, (Median, IQR)	1 (1, 2)	1 (1, 2)	0.817
mMRC scores, n (%)			0.734
0-1	59 (53.2)	228 (51.4)	
≥2	52 (46.8)	216 (48.6)	
Comorbidities, n (%)			
Chronic heart disease	3 (2.7)	21 (4.7)	0.442
Hypertension	5 (4.5)	28 (6.3)	0.473
Lung cancer	3 (2.7)	5 (1.1)	0.202
Diabetes	1 (0.9)	10 (2.3)	0.702
Bronchiectasis	8 (7.2)	32 (7.2)	1.000
Exacerbations in the past year, (Median, IQR)	0 (0, 1)	0 (0, 1)	0.816

Exacerbations in the past years, n (%)			0.828
0	60 (54.1)	227 (51.1)	
1	25 (22.5)	111 (25.0)	
≥20	26 (23.4)	106 (23.9)	
Hospitalizations in the past year, (Median, IQR)	0 (0, 1)	0 (0, 1)	0.632
Hospitalizations in the past year, n (%)			0.653
0	72 (64.9)	298 (67.1)	
≥1	39 (35.1)	146 (32.9)	

Abbreviations: BMI, Body Mass Index; COPD, Chronic Obstructive Pulmonary Disease; CAT, COPD Assessment Test; FEV1 %pred, Forced Expiratory Volume in one second percentage predicted; FEV1, Forced Expiratory Volume in one second; FVC, Forced Vital Capacity; ICS, Inhaled Corticosteroid; IQR, InterQuartile Range; LAMA, Long-Acting Muscarinic Antagonist; LABA, Long-Acting β2-Agonist; mMRC, modified Medical Research Council; SAMA, Short-Acting Muscarinic Antagonist; SABA, Short-Acting β2-Agonist.

Supplement table 4. The treatment responses among different inhalation therapies of the patients during one year of follow-up.

Variables	LAMA (N=108)	LABA+LAMA (N=77)	LABA+ICS (N=146)	LABA+LAMA+ICS (N=124)	P - values
Exacerbations during one year, (Median, IQR)	0 (0, 0)	0 (0, 0)	0 (0, 0)	0 (0, 0)	0.221
Exacerbations, n (%)					0.171
No	95 (88.0)	63 (81.8)	118 (80.8)	95 (76.6)	
Yes	13 (12.0)	14 (18.2)	28 (19.2)	29 (23.4)	
Frequent exacerbations, n (%)					0.798
No	102 (94.4)	71 (92.2)	136 (93.2)	113 (91.1)	
Yes	6 (5.6)	6 (7.8)	10 (6.8)	11 (8.9)	
Hospitalizations during one year, (Median, IQR)	0 (0, 0)	0 (0, 0)	0 (0, 0)	0 (0, 0)	0.311
Hospitalizations, n (%)					0.296
No	101 (93.5)	70 (90.9)	133 (91.1)	107 (86.3)	
Yes	7 (6.5)	7 (9.1)	13 (8.9)	17 (13.7)	
All-cause of mortality, n (%)					0.109
Yes	3 (2.8)	0 (0)	0 (0)	1 (0.8)	
No	105 (97.2)	77 (100)	146 (100)	123 (99.2)	

Abbreviations: ICS, Inhaled Corticosteroid; IQR, InterQuartile Range; LAMA, Long-Acting Muscarinic Antagonist; LABA, Long-Acting β2-Agonist.

Supplement table 5. Multivariate analysis for future exacerbation.

Variables	Exacerbation		Frequent exacerbation		Hospitalization		All-cause of mortality	
	**OR (95%CI)**	**P-value**	**OR (95%CI)**	**P-value**	**OR (95%CI)**	**P-value**	**OR (95%CI)**	**P-value**
Therapy								
LAMA	Reference		Reference		Reference		N/A	
LABA+LAMA	1.82 (0.77-4.31)	0.176	1.64 (0.48-5.57)	0.431	1.63 (0.52-5.09)	0.399	N/A	N/A
LABA+ICS	1.80 (0.85-3.79)	0.123	1.34 (0.45-3.98)	0.596	1.46 (0.54-3.94)	0.458	N/A	N/A
LABA+LAMA+ICS	1.83 (0.86-3.88)	0.117	1.48 (0.50-4.38)	0.478	2.02 (0.77-5.29)	0.154	N/A	N/A

Notes: Factors in the logistic model: Therapy, prescription outcome, age, sex, BMI, smoke history, biofuel exposure, FEV1%, FEV1/FVC, CAT scores, exacerbations in the past year, and Comorbidities. N/A, not applicable.

Abbreviations: BMI, Body Mass Index; COPD, Chronic Obstructive Pulmonary Disease; CAT, COPD Assessment Test; FEV1 %pred, Forced Expiratory Volume in one second percentage predicted; FEV1, Forced Expiratory Volume in one second; FVC, Forced Vital Capacity; ICS, Inhaled Corticosteroid; LAMA, Long-Acting Muscarinic Antagonist; LABA, Long-Acting β2-Agonist; mMRC, modified Medical Research Council; OR, Odds Ratio, CI, Confidence Interval.

Supplement table 6. Multivariate analysis for future exacerbation.

Variables	Exacerbation		Frequent exacerbation		Hospitalization		Mortality	
	**OR (95%CI)**	**P-value**	**OR (95%CI)**	**P-value**	**OR (95%CI)**	**P-value**	**OR (95%CI)**	**P-value**
Therapy								
LABA+LAMA	Reference		Reference		Reference		N/A	
LAMA	0.55 (0.23-1.31)	0.176	0.61 (0.18-2.08)	0.431	0.61 (0.20-1.91)	0.399	N/A	N/A
LABA+ICS	0.99 (0.46-2.15)	0.982	1.82 (0.27-2.54)	0.731	0.89 (0.32-2.51)	0.830	N/A	N/A
LABA+LAMA+ICS	1.01 (0.47-2.15)	0.987	0.90 (0.30-2.71)	0.858	1.24 (0.46-3.30)	0.672	N/A	N/A

Notes: Factors in the logistic model: therapy, prescription outcome, age, sex, BMI, smoke history, biofuel exposure, FEV1%, FEV1/FVC, mMRC, exacerbations in the past year, and Comorbidities. N/A, not applicable.

Abbreviations: BMI, Body Mass Index; COPD, Chronic Obstructive Pulmonary Disease; CAT, COPD Assessment Test; FEV1 %pred, Forced Expiratory Volume in one second percentage predicted; FEV1, Forced Expiratory Volume in one second; FVC, Forced Vital Capacity; ICS, Inhaled Corticosteroid; LAMA, Long-Acting Muscarinic Antagonist; LABA, Long-Acting β2-Agonist; mMRC, modified Medical Research Council; OR, Odds Ratio, CI, Confidence Interval.
